# Chlorhexidine-impregnated dressing for the prophylaxis of central venous catheter-related complications: a systematic review and meta-analysis

**DOI:** 10.1186/s12879-019-4029-9

**Published:** 2019-05-16

**Authors:** Li Wei, Yan Li, Xiaoyan Li, Lanzheng Bian, Zunjia Wen, Mei Li

**Affiliations:** Children’s Hospital of Nanjing Medical University, No.72 Guangzhou road, Gulou district, Nanjing, Jiangsu province China

**Keywords:** Chlorhexidine, CRBSI, Central venous catheter, Nursing care, Nosocomial infection

## Abstract

**Background:**

Several randomized controlled trials (RCTs) evaluated the role of Chlorhexidine-impregnated dressing for prophylaxis of central venous catheter (CVC) related complications, but the results remained inconsistent, updated meta-analyses on this issue are warranted.

**Methods:**

A meta-analysis on the RCTs comparing Chlorhexidine-impregnated dressing versus other dressing or no dressing for prophylaxis of central venous catheter-related complications was performed. A comprehensive search of major databases was undertaken up to 30 Dec 2018 to identify related studies. Pooled odd ratio (OR) and mean differences (MDs) with 95% confidence intervals (CI) were calculated using either a fixed-effects or random-effects model. Subgroup analysis was performed to identify the source of heterogeneity, and funnel plot and Egger test was used to identify the publication bias.

**Results:**

A total of 12 RCTs with 6028 patients were included. The Chlorhexidine-impregnated dressings provided significant benefits in reducing the risk of catheter colonization (OR = 0.46, 95% CI: 0.36 to 0.58), decreasing the incidence of catheter-related bloodstream infection (CRBSI) (OR = 0.60, 95% CI: 0.42 to 0.85). Subgroup analysis indicated that the Chlorhexidine-impregnated dressings were conducive to reduce the risk of catheter colonization and CRBSI within the included RCTs with sample size more than 200, but the differences weren’t observed for those with sample less than 200. No publication bias was observed in the Egger test for the risk of CRBSI.

**Conclusions:**

Chlorhexidine-impregnated dressing is beneficial to prevent CVC-related complications. Future studies are warranted to assess the role and cost-effectiveness of Chlorhexidine-impregnated dressings.

## Background

It’s very common that clinically indwelling central venous catheter (CVC) to meet the treatment needs, especially for patients admitted to intensive care unit (ICU) [1, 2]. The insertion of CVC provides credible pathway to meet the needs of rapid rehydration, the use of vasoactive drugs, hemodynamic monitoring and parenteral nutrition support, etc. [3]. However, the catheter-related bloodstream infection (CRBSI) may accompany with the use of CVC-related devices [4]. It’s been reported that the rate of CRBSI ranges from 0.8 to 0.2 per 1000 central-line catheter days [5, 6]. Besides, it’s well known that CRBSI leads to increased use of antibiotics, longer length of hospital stay, excessive burdens of healthcare costs and even higher mortality [7–9]. Therefore, effective strategies to prevent CRBSIs are essential to improve the prognosis of patients with CVC.

Currently, many CLABSI care bundles have been applied to prevent CRBSIs, which include highlighting hand hygiene, the maximum full-barrier precautions during the insertion process, and skin antisepsis etc. [10, 11]. In recent years, the use of Chlorhexidine for CRBSIs prevention has drawn numerous attentions from clinically health care providers. Many studies have reported the applications of Chlorhexidine in different ways, such as Chlorhexidine for bathing, disinfection, oral care and dressing-containing. Based on literature review, we found that several randomized controlled trials (RCTs) evaluated and reported the role of Chlorhexidine-impregnated dressing for prophylaxis of CVC-related complications, but the results remained inconsistent and even controversial. Furthermore, currently the systematic reviews on the role of Chlorhexidine-impregnated dressing for prophylaxis of CVC-related complications are quite few. Besides, there are several new RCTs on this issue has been reported. Therefore, it’s necessary to conduct an updated meta-analysis to evaluate the role of Chlorhexidine-impregnated dressing for prophylaxis of CVC-related complications, thereby providing more evidences for the management of CVC.

## Methods

This present systematic review was conducted and reported in compliance with the Preferred Reporting Items for Systematic reviews and Meta-Analyses (PRISMA) statement [12].

### Search strategies

To identify potential eligible RCTs, a systematic literature search was conducted in following databases: PubMed, EMBASE, Science Direct, Cochrane Central Register of Controlled Trials, China National Knowledge Infrastructure and Wanfang Database (from inception to 30 Dec 2018). Following search terms were used according to the rule of each database: “Chlorhexidine”, “dressing”, “sponge”, “bloodstream”, “infection”, “colonization”. The reference lists of articles were retrieved by two authors (L W and X L) and the authors of included RCTs were contacted to obtain additional data if necessary. Furthermore, the ClinicalTrials.gov and the WHO International Clinical TrialsRegistry Platform were manually searched for unpublished, planned or ongoing trial reports. And also the OpenGrey was manually searched to identify grey literature.

### Criteria for included studies

RCTs comparing Chlorhexidine-impregnated dressing versus other dressing or no dressing for prophylaxis of CVC-related complications were included irrespective of the language of publication, publication status, year of publication, or sample size.

### Data extraction

Two authors (L W and Y L) independently evaluated the titles, abstracts and full-text of identified studies, any controversy was resolved by further discussion. The following data were collected for each included study whenever it’s available: authors, publication year, country of origin, study population, numbers of participants, type of inserted catheter, Chlorhexidine-impregnated dressing intervention, definition of catheter colonization and CRBSI, outcome variables and study conclusions. The original authors were further contacted by email if there were something unclear. Two authors (L W and Y L) independently reviewed the included RCTs, and extracted and collected related data. All disagreements were resolved by further discussions.

### Quality assessment

The Cochrane Collaboration’s risk of bias tool [13] was used by two authors (L W and Y L) independently to evaluate the methodological quality and risk of bias of included RCTs; any disagreement was resolved by discussion and consensus.

### Data analysis

The software RevMan 5.3 was used to perform statistical analyses in this present study. Binary outcomes were presented as Mantel–Haenszel-style odds ratios (ORs) with 95% confidence interval (95%CI), and continuous outcomes were reported as mean differences (MDs). The presence of heterogeneity among trials was assessed by using chis-square test (*p* < 0.05 denoted statistical significance in the analysis of heterogeneity), whereas the degree of heterogeneity was assessed by I^2^ statistic with a threshold of 50%, a random-effect or fix-effect model was used according to the degree of heterogeneity. The source of heterogeneity was detected by subgroup analysis, and the interaction was significant if the *P* value < 0.05 based on the sample size, effect size and 95% CI of each subgroup. Publication bias was evaluated by using funnel plots, and the asymmetry was assessed by conducting Egger regression test. Furthermore, we conducted sensitivity analyses to identify the impact of single study on the whole synthesized results. *P* < 0.05 was considered that the difference was statistically significant.

## Results

### Characteristics of included studies

**Fig. 1:**
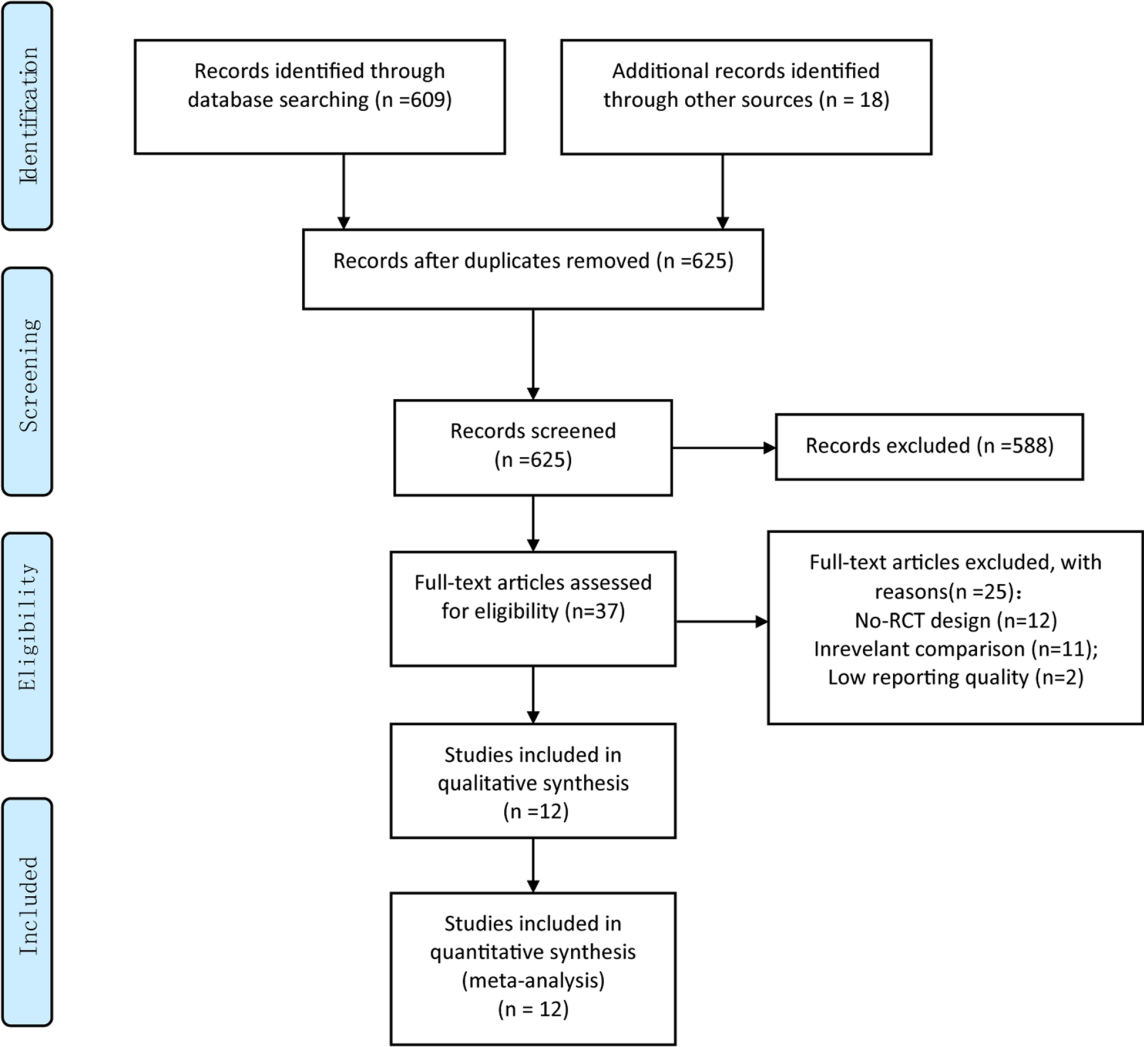
Flow chart of study selection

A total of 609 references were obtained from the initial electronic database searches. Eighteen additional references were identified from other sources. After de-duplication, 625 references were screened, and 588 reference were excluded after first screening on the title and abstract, thus 37 references underwent further full-text screening. Based on the inclusion and exclusion criteria, finally 12 RCTs [14–25] were included. Figure 1 presents the PRISMA flowchart for study selection.

Table 1 shows the characteristics of 12 included RCTs [14–25]. Of the 12 included RCTs, a total of 6028 patients were involved, with 3242 patients for Chlorhexidine-impregnated dressing intervention, and 2786 patients for other intervention respectively. The included RCTs were conducted in several different countries, one RCT [17] focused on the population of neonates, and one [18] focused on pediatrics, the resting RCTs were all conducted on adults. For the type of CVC, tunneled and non-tunneled CVC were both reported among the included RCTs. For Chlorhexidine intervention, the Chlorhexidine-impregnated dressings were generally applied after catheterization and changed every 3 days. For observed outcomes, five studies [14, 18, 20, 21, 25] failed to detect the effects of Chlorhexidine-impregnated dressings on reducing the incidence of CRSBI or colonization, while the resting seven RCTs [15–17, 19, 22–24] favored that the Chlorhexidine-impregnated dressings were beneficial to reduce the risk of CRSBI.

**Table 1:** the characteristics of included studies

Author(year)	Country	Population	Numbers of participants(Chlorhexidine / Control)	Catheter Type	Chlorhexidine-impregnated dressing Intervention	Definition of Catheter Colonization	Definition of CRBSI	Conclusion
Arvaniti 2012	Greece	ICU patients who required a CVC for ≥3 days	150/156	CVC	after the first 24 h of catheterization, a Biopatch was placed underneath the transparent dressing, And the Biopatch was changed every 3 days,	Quantitative CVC tip culture with > 1000 CFU/mL and no systemic signs of sepsis	Quantitative CVC tip culture with > 1000 CFU/mL with systemic signs of sepsis	Chlorhexidine-impregnated sponges and Oligon catheters as single preventive measures did not reduce catheter colonization or catheter-related infections.
Biehl 2016	Germany	Patients undergoing chemotherapy with an expected duration of chemotherapy- induced neutropenia of ≥5 days and an expected CVC use of ≥10 days	307/306	Non-tunneled CVC	Dressings were applied within 2 h of CVC placement and changed every 3 ± 1 days.	Not availabe	The results from blood and CVC tip cultures	The application of chlorhexidine containing catheter securement dressings reduces the incidence of definite or probable CRBSI in neutropenic patients.
Chambers 2005	New Zealand	Adult patients undergoing chemotherapy in a haematology unit.	58/54	Long-term, tunneled and cuffed CVC	The Chlorhexidine impregnated dressing were applied to the exit site as soon the oozing had stopped following intravascular catheter insertion, and changed as needed or weekly.	Not available	Fever and positive blood cultures without alternative infection source and catheter tip culture with > 15 colonies of the same organism	Chlorhexidine dressings reduced the incidence of exit-site/tunnel infections of indwelling tunnelled intravascular catheters without prolonging catheter survival in neutropenic patients
Garland 2001	USA	ICU neonates with CVC expected to remain in place a minimum of 48 h	335/370	CVC and tunneled (Broviac) CVC	The Chlorhexidine impregnated dressing were applied after catheterization and changed for every 7 days or as needed.	Semiquantitative catheter colony count > 15 CFU	Clinical infection with same organism isolated from catheter tip and blood	The Chlorhexidine impregnated dressing is effective in protect against the catheter-tip colonization.
Gereker 2017	Turkey	Pediatric hematologyoncology (PHO) population over 2 months of age with expected CVC duration over 48 h	14/13	CVC	Care bundle with Chlorhexidine impregnated dressing being used	Not availale	Blood culture	There was no difference between the two groups with chlorhexidine dressing or advanced dressings in terms of CRBSI development.
Levy 2005	Israel	Cardiac ICU pediatrics requiring CVC for minimum of 48 h	74/71	Short-term, nontunneled CVC	The Chlorhexidine impregnated dressing were applied after catheterization and changed whenever needed.	> 15 CFU by the roll-plate technique, no signs of infection	Bacteremia with isolation of the same organism from CVC tip and blood	The Chlorhexidine impregnated dressing is safe and significantly reduces the rates of CVC colonization in infants and children after cardiac surgery.
Pedrolo 2014	Brazil	Adult ICU patients	43/42	CVC	chlorhexidine antimicrobial dressing was changed every 7 days	Not available	Infection variables: temperature > 38 °C, systolic blood pressure < 90 mmHg, oliguria< 20 ml/h, tenderness, pain or swelling on palpation, hyperemia, cyanosis or discharge at the catheter opening; And further verified by blood culture or a culture of the catheter tip	The chlorhexidine antimicrobial dressing is not effective in reducing primary bloodstream infection when compared to the gauze and tape dressing.
Roberts 1998	Australia	Adult ICU patients receiving CVC over a 7-week period	17/16	CVC	Chlorhexidine impregnated dressing was changed every fifth day or as needed.	Same organism from CVC tip and exit site, no clinical infection	Clinical infection with same organism isolated from catheter tip (and/or exit site) and blood	No statistical difference was found between the two groups with regard to CVC or exit-site colonisation.
Ruschulte 2008	Germany	Adults receiving chemotherapy with catheter expected for minimum of 5 d	300/301	Triple-lumen CVC	The Chlorhexidine impregnated dressing were applied after catheterization	Not available	Clinical evidence of infection and time-to-positivity method used with CVC and peripherally drawn blood cultures	The use of chlorhexidine-impregnated wound dressings significantly reduced the incidence of CVC-related infections in patients receiving chemotherapy.
Timsit 2009	France	Adult ICU patients requiring catheter minimum of 48 h	817/819	CVC and/ or arterial catheter	The Chlorhexidine impregnated dressing was changed 24 h after catheter insertion (day 1) and then every 3 days in the 3-day group and every 7 days in the 7-day group	Quantitative CVC tip culture ≥1000 CFUs/mL	Clinical infection without alternative source, peripheral blood drawn immediately prior to or within 48 h following catheter removal and quantitative catheter tip culture isolating the same organism, or confirmed using differential time to positivity test	The use of Chlorhexidine impregnated dressings decreased the risk of major catheter- related infections by 60% despite a low baseline infection rate.
Timsit 2012	France	ICU patients with vascular catheters inserted for an expected duration of more than 48 h	938/476	CVC	The dressings were changed 24 h after catheter insertion (Day 1) then every 3 or 7 days according to standard practice in each ICU	Quantitative CVC tip culture > 1000 CFU/mL and no systemic signs of sepsis	Correlation between peripheral blood culture and quantitative tip culture without other likely source	The Chlorhexidine-impregnated gel dressings decrease by 60% the risk of CRBSI in the ICU.
Yu 2015	China	Adult internal ICU patients	189/162	CVC	Chlorhexidine impregnated dressing was changed every 7 days or as needed.	Not available	Infection variables: blood cultures or catheter tip cultures	The Chlorhexidine impregnated dressing cannot effectively reduce the incidence of CLABSI, and the cost is higher, but it can effectively reduce the number of dressing changes and save labor costs

*ICU* intensive care unit, *CVC* Central venous catheter, *CLABSI* catheter-related bloodstream infection

### Risk of bias evaluation

**Fig. 2:**
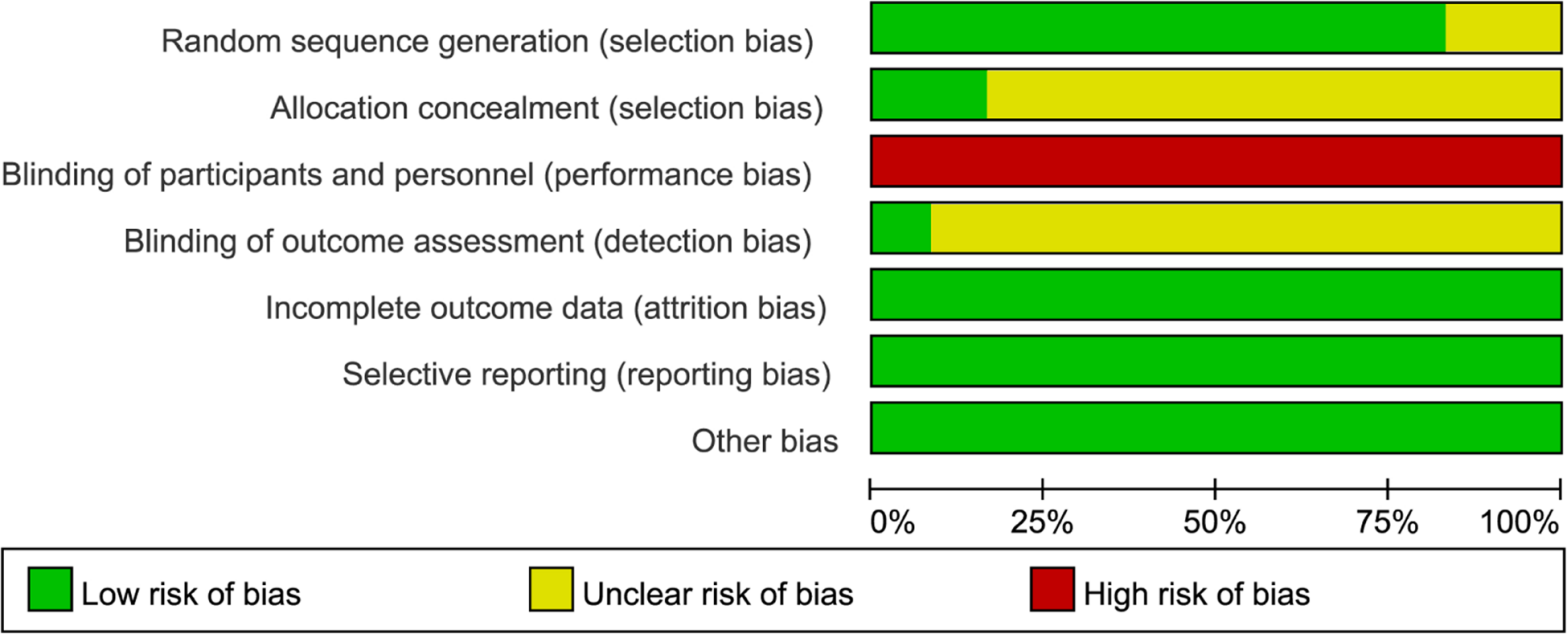
Risk of bias graph

**Fig. 3:**
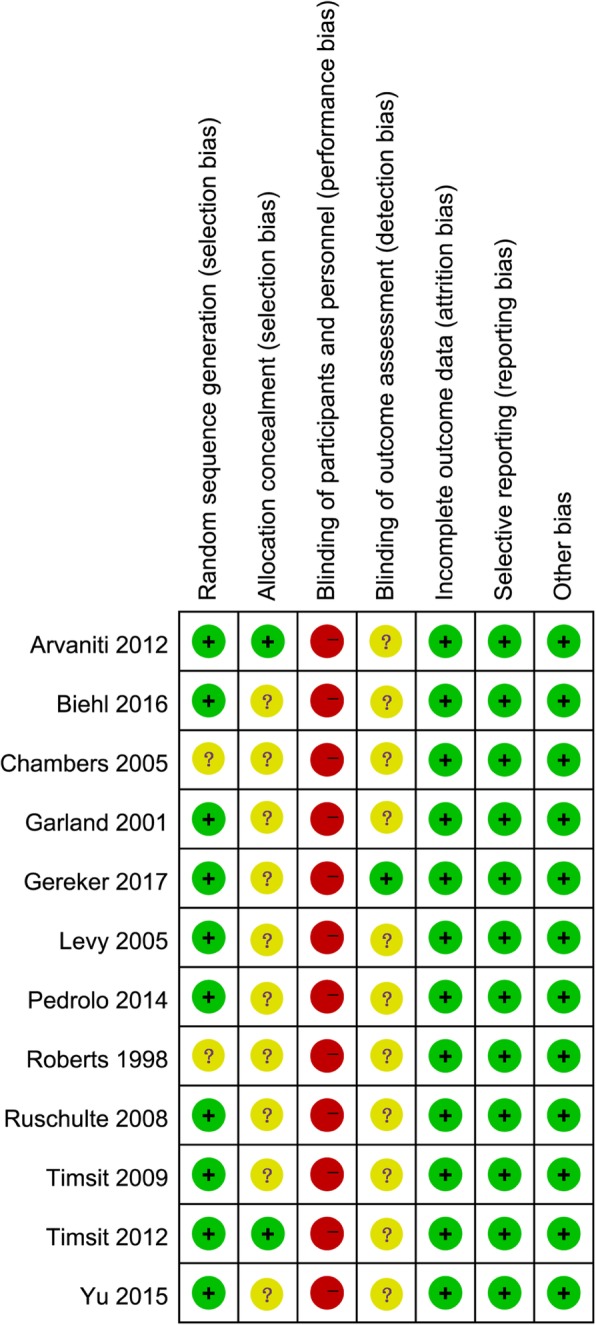
Risk of bias summary

Figures 2 and 3 indicate the risk of bias for each included study. Briefly, all included RCTs mentioned randomization in their reports, but two RCTs [16, 21] failed to report the methods to generate random sequence. Only two studies [14, 24] reported the methods to perform allocation concealment. All included studies were rated as high risk of performance bias as they were unable to blind the personnel or participant about the intervention allocated. Only one RCT [18] reported the blind design during the outcome assessment, thereby it was rated as low risk of detection bias. No other kinds of biases were found.

### Effects of interventions

**Fig. 4:**
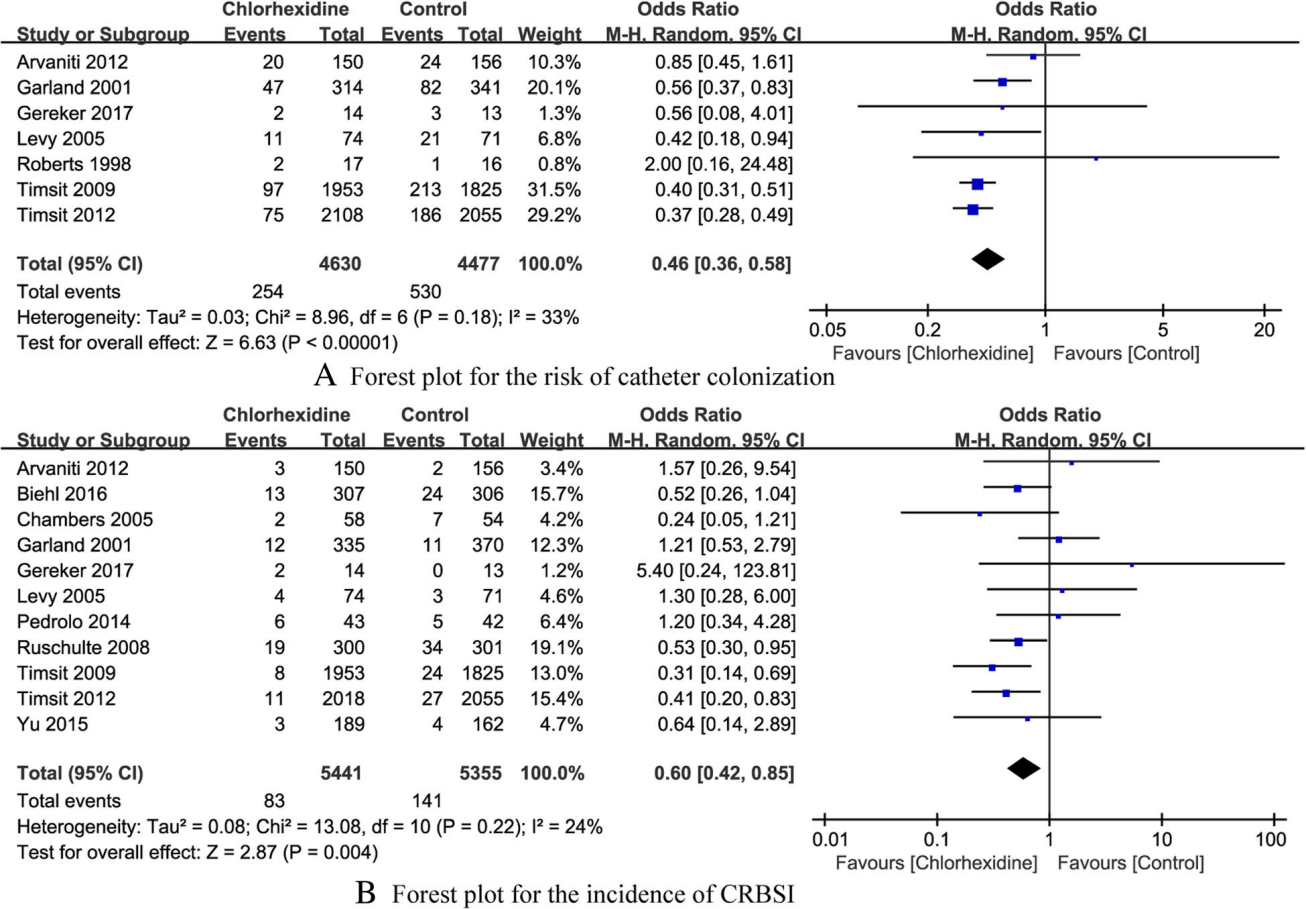
The forest plot for different outcomes

*The risk of catheter colonization* A total of seven RCTs [14, 17–19, 21, 23, 24] reported the risk of catheter colonization. The summary OR on the risk of catheter colonization between Chlorhexidine and control group was 0.46(95% CI, 0.36 to 0.58), without evident heterogeneity (*P* < 0.18, I^2^ = 33%) (Fig. 4a). The results indicated that Chlorhexidine-impregnated dressings was beneficial to reduce the risk of catheter colonization.

*The incidence of CRBSI* A total of 11 RCTs [14–20, 22–25] reported the incidence of CRBSI. The summary OR on the incidence of CRBSI between Chlorhexidine and control group was 0.60(95% CI: 0.42 to 0.85), without evident heterogeneity (*P* = 0.22, I^2^ = 24%) (Fig. 4b). The results indicated that the Chlorhexidine-impregnated dressings were conducive to reduce the incidence of CRBSI.

### Subgroup analysis

**Fig. 5:**
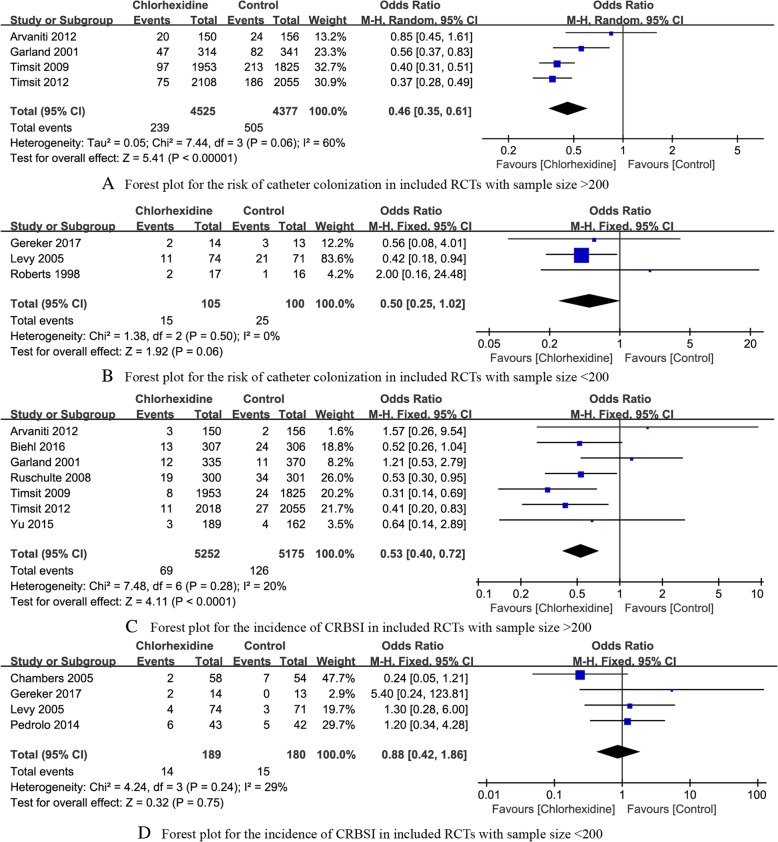
The forest plot for outcomes stratified by sample size more or less than 200

We conducted subgroup analysis stratified by study size less or more than 200 patients, and the interactions were significant on the risk of catheter colonization and CRBSI when study size less or more than 200 patients (all *P* < 0.05), indicating that study size less or more than 200 patients is a potential influencing factor. As Fig. 5 showed, the Chlorhexidine-impregnated dressings provided more benefits in reducing the risk of catheter colonization and CRBSI among the included RCTs with sample size more than 200, but the differences weren’t observed among the included RCTs with sample size less than 200.

### Publication bias

**Fig. 6:**
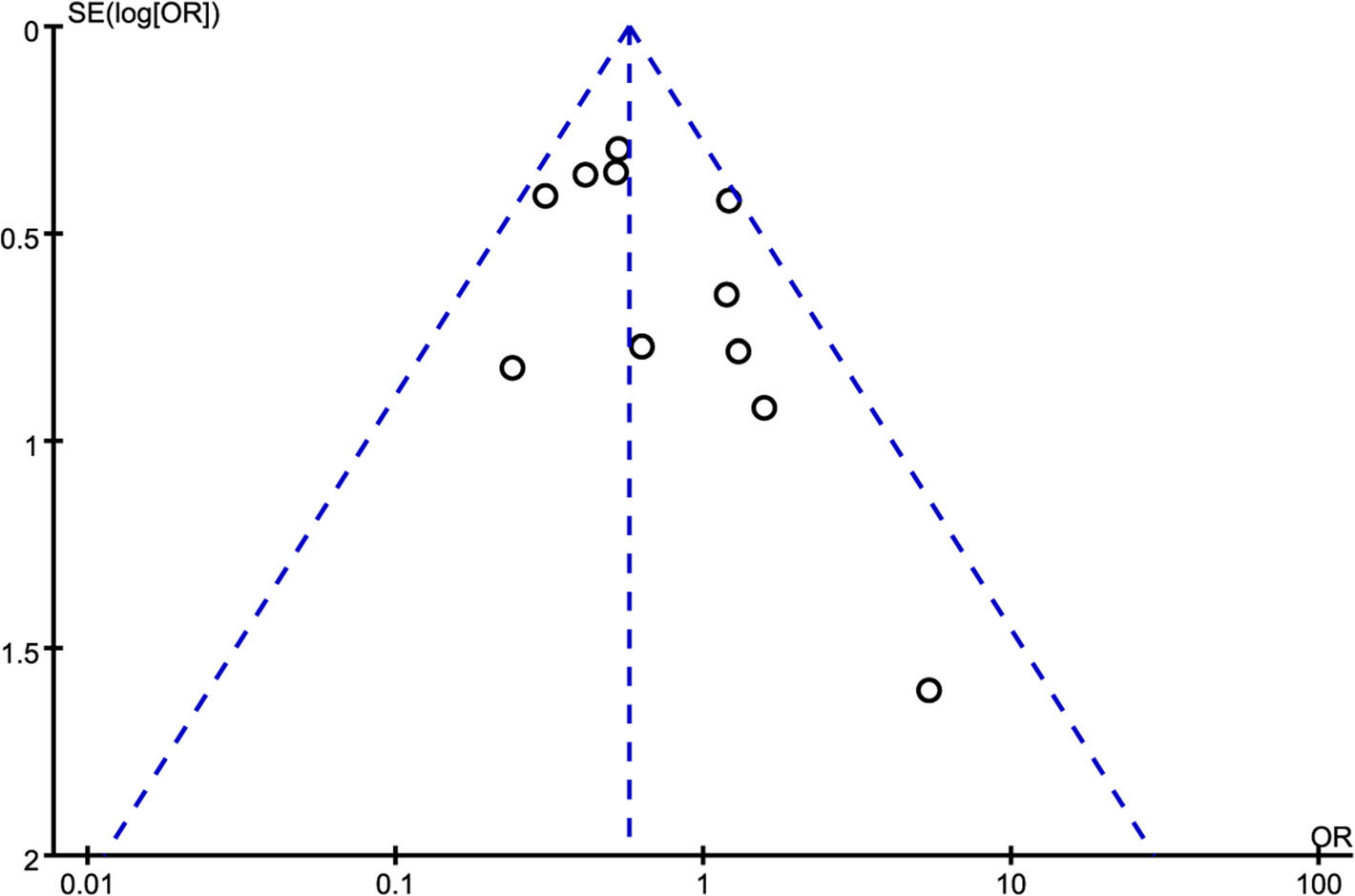
The funnel plot for the risk of CRBSI

The funnel plot on the risk of catheter colonization is presented in Fig. 6, and even though the funnel plot was asymmetrical as it looked, but no publication bias was detected in the risk of CRBSI by Egger test (*P* = 0.071).

### Sensitivity analysis

We excluded RCTs on each result one by one to see that if the overall results changed, and we found that the overall results weren’t changed by exclusion of any included RCTs.

## Discussion

With 12 RCTs included, the results of this meta-analysis indicate that the use of Chlorhexidine-impregnated dressing is beneficial to reduce the risk of catheter colonization and CRBSI for patients with CVC, it’s an effective anti-infection strategy in preventing CRBSI. Our results are consistent with the previous findings of meta-analyses [26, 27], but with more RCTs included for synthesized analysis, our results do provide more strength in increasing the statistical effectiveness. As such, this study further supports the use of Chlorhexidine-impregnated dressing for prophylaxis of CVC-related complications.

Currently, several clustering care strategies in nursing care have been utilized to prevent CRBSI, such as the maximum sterile barriers, choosing appropriate location for insertion, disinfection of skin with Chlorhexidine, and daily assessment of the need for catheter removal etc. However, the results of published RCTs on Chlorhexidine-impregnated dressing as a preventive strategy for CRBSI remain controversial. Based on literature reviews, the incidences of CRBSI varied greatly among different areas. The National Healthcare Safety Network reported that the CRBSI rates were 1.0‰~ 1.4‰ in adult ICUs of developed countries in 2010 [28], whereas International Nosocomial Infection Control Consortium conducted a survey of 36 developing countries in Latin America, Asia, Africa and Europe, and it reported that the CRBSI rate was 6.8 ‰ [29]. The rate of CRBSI in China was 2.9 ‰~ 11.3‰ [30]. However, the overall rate of CRBSI in this present study is 11.5‰, which is higher than that of previous reports. Nevertheless, the rate of CRBSI in Chlorhexidine group is 15.2‰, yet the rate of CRBSI in control group is 26.3‰, a significant difference was detected between this two groups. Therefore, the application of Chlorhexidine-impregnated dressing is conducive to reduce the risk of CRBSI in patients with CVC.

Chlorhexidine gluconate is one kind of cationic surfactants, it’s commonly used for disinfecting skin or mucosal tissues clinically, the mechanism of Chlorhexidine gluconate for disinfection is that destroying the permeation barrier on bacterial cell membrane. At present, there are two kinds of Chlorhexidine dressings used clinically, one is one-piece, that is, the dressing itself is self-contained with Chlorhexidine, the other is a separate type, which needs to be covered with Chlorhexidine cotton, plus further transparent dressing covering. Pfaff [31] compared the effectiveness of a new one-piece occlusive dressing that incorporated Chlorhexidine gluconate with that of a dressing plus a Chlorhexidine gluconate patch, found that the new dressing provided more advantages in reducing the incidence of CRBSI, improving nurses’ satisfaction and saving medical cost. Additionally, since the dressings containing Chlorhexidine only need to be changed every 7 days, the frequency of dressing change reduces significantly when compared to the routine dressings requiring change every 3 days, thereby reducing the risk of infection and workload of nursing care [32, 33].

The definition, importance and potential relationship of colonization and CRBSI must be considered. Generally, the catheter is considered as being colonized when the culture of tip yield ≥15 colony-forming units of the same colony type, whereas CRBSI is defined as the presence of the same organism (identical species and anti-microbial susceptibility pattern) in a colonized PICC and in blood cultures from the same event [34]. Meanwhile, it’s been reported that cutaneous colonization is related to CRBSI [35]. There are some intersection in the definition of colonization and CRBSI, and the most included RCTs have both reported the colonization and CRBSI, yet the incidence of colonization and CRBSI varied greatly among the included RCTs, there is a possibility that making mistakes on mixing colonization and CRBSI, which is a potential source of result heterogeneity.

It should be highlighted that there are many factors influencing the incidence of CRBSI, which includes the site selection of CVC placement, the operation of catheterization, the maintenance after catheterization etc. [36–38] The Chlorhexidine-impregnated dressing on the puncture site is only one related factors, more nursing care bundles must be considered in preventing CRBSI. Previous studies [39–41] have shown that the incidence of CRBSI is highest in the case of femoral vein catheterization, while the subclavian vein is the site with lowest incidence of CLABSI, and the internal jugular vein is the second. The aseptic techniques and the proficiency of operator during the catheterization are also closely related to CRBSI, repeated punctures can cause damage to the vessel wall and subcutaneous tissue, thereby increasing infection risk attributed to bacterial invasion [42, 43]. We attempted to conduct sub-group analysis according to catheter site, but the data on the catheter site among the included RCT were not fully available, future studies should focus on the role of catheter site and related nursing bundles in the management of CVC.

The cost of Chlorhexidine-impregnated dressing must also be concerned. It was reported that chlorhexidine-impregnated sponge use saved $197 by preventing infection per patient with the 3-day chlorhexidine-impregnated sponge dressing change strategy, and $83 with the 7-day standard dressing change strategy [44]. We attempted to compare the costs of Chlorhexidine-impregnated dressing with other dressing, however, only one RCT [25] reported this outcome, and this study [25] found that the use of Chlorhexidine transparent dressing could not save the direct economic cost of dressing, nor reduce length of ICU stay to save the indirect economic costs, but it could effectively reduce the frequency of dressing changes to ease the workload of nursing staff. Future studies are warranted to provide more insights into the economic evaluation on the use of Chlorhexidine-impregnated dressing.

Several limitations in this meta-analysis must be considered. Firstly, we didn’t use mesh terms in our search strategy or ask for help from a librarian developing the search strategy, therefore, there was possibility that some article might be missed in our initial search. Secondly, considering the nature of intervention, it’s rather difficult to blind the research personnel and outcome assessment, none of included RCTs was truly double blind design, hence the risk of bias is inevitable. And the blood culture was conducted in elected patients only among the included RCTs, this might also introduce bias. Thirdly, the rates of CRBSI among included RCTs varied greatly with a range of 0 to 11.3%, it might be related to the differences in clinical nursing practice and guidelines. Fourthly, the Egger test for the detection of publication bias was potentially underpowered given the small sample size, a non-significant Egger’s test did not necessarily suggest lack of asymmetry in the Funnel plot, therefore, this results should be treated with cautions. Finally, we only made post-hoc subgroup analyses stratified by sample size, but not by insertion location, type of Chlorhexidine-impregnated dressing, the frequency of dressing changes etc. due to the data limitation, the publication bias on the risk of catheter colonization remained unclear, future studies addressing the role of Chlorhexidine-impregnated dressing with combined consideration to those related factors are warranted.

## Conclusions

In conclusions, the application of Chlorhexidine-impregnated dressing is effective in reducing the risks of catheter colonization and CRBSI for patients with CVC, which is beneficial to the prognosis of patients and it may be potentially worthy of clinical use. Future studies are needed to evaluate the cost-effectiveness of Chlorhexidine-impregnated dressing use and other related preventative strategies. Moreover, further stratified analysis of Chlorhexidine-impregnated dressing use and CRBSI-related factors are needed to elucidate the optimal prophylaxes for CRBSI.

## Abbreviations

CIs: confidence intervals
CRBSI: catheter-related bloodstream infection
CVC: central venous catheter
ICU: intensive care unit
MDs: mean differences
ORs: odd ratios
PRISMA: preferred reporting items for systematic reviews and meta-analyses
RCTs: randomized controlled trials
